# Targeting the liver to treat the eye

**DOI:** 10.15252/emmm.202217285

**Published:** 2023-02-27

**Authors:** Berna Seker Yilmaz, Paul Gissen

**Affiliations:** ^1^ Genetics and Genomic Medicine Department, Great Ormond Street Institute of Child Health University College London London UK; ^2^ National Institute of Health Research, Great Ormond Street Biomedical Research Centre London UK; ^3^ Metabolic Medicine Department Great Ormond Street Hospital for Children NHS Foundation Trust London UK

**Keywords:** Genetics, Gene Therapy & Genetic Disease

## Abstract

Over the last two decades, gene therapy has given hope of potential cure for many rare diseases. In the simplest form, gene therapy is the transfer or editing of a genetic material to cure a disease via nonviral or viral vehicles. Gene therapy can be performed either *in vivo* by injecting a vector carrying the gene or tools for gene editing directly into a tissue or into the systemic circulation, or *ex vivo* when patient cells are genetically modified outside of the body and then introduced back into the patient (Yilmaz *et al*, 2022). Adeno‐associated viral vectors (AAV) have been the vectors of choice for *in vivo* gene therapy. There has been a lot of promising research on the development of novel tissue and cell‐specific serotypes in order to improve efficacy and safety for clinical applications (Kuzmin *et al*, 2021). In this issue of EMBO Molecular Medicine, Boffa and colleagues present a novel AAV‐based liver‐directed gene therapy for ornithine aminotransferase deficiency.

## Graphical abstract

P Gissen and B Şeker Yilmaz discuss novel liver‐directed gene therapy for gyrate atrophy of the choroid and retina due to ornithine aminotransferase deficiency as reported by N Brunetti‐Pierri and colleagues, in this issue of *EMBO Mol Med*.

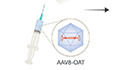

The eye has been a prime target for gene therapies, and there are over 100 gene therapy clinical trials, mostly using AAV, registered for ocular diseases. In 2017, voretigene neparvovec (VN)‐rzyl (Luxturna^®^ Spark Therapeutics Inc.) became the first US Food and Drug Administration (FDA)‐approved ocular gene therapy, and later, in 2018, it was approved by the European Medicines Agency (EMA, voretigene neparvovec [Luxturna^®^Novartis]), for the treatment of biallelic RPE65 mutation‐associated inherited retinal dystrophy with viable retinal cells (Maguire *et al*, 2009; Russell *et al*, 2017). These approvals represented hugely important milestones in the evolution of gene therapy.

The liver is a key metabolic organ with its most prevalent cell, the hepatocyte, hosting the majority of metabolic pathways. The first triumph in the field of liver‐directed gene therapy was completed with the approval of the world's first product, valoctocogene roxaparvovec (Roctavian) for hemophilia A. Etranacogene dezaparvovec (AMT‐061) for hemophilia B is also in the final stages of approval. In hemophilia, the aim of therapy is for the transduced hepatocytes to generate and secrete the missing clotting factor proteins. A small number of transduced hepatocytes may secrete a sufficient amount of the deficient protein to a make significant clinical difference. The success in hemophilia gene therapy research led to the development of gene therapy trials in disorders where endogenous hepatocyte pathways need to be restored such as ornithine transcarbamylase (OTC) deficiency, phenylketonuria, glycogen storage disease 1a (GSD1a), Crigler–Najjar syndrome, and many others (Baruteau *et al*, 2017; Nathwani *et al*, 2022). Various types of AAV vectors are considered hepatotropic and can efficiently mediate gene transfer in postmitotic hepatocytes; thus, they have emerged as the leading candidates for liver‐targeted gene therapy (Kuzmin *et al*, 2021; Yilmaz *et al*, 2022). One of the challenges of using nonintegrating AAV vectors for liver‐targeted gene therapy is that their expression gets progressively reduced during the period of rapid liver growth. Thus, the administration of the AAV at later age provides a potential advantage to the long‐term persistence of the therapeutic effect.

Ornithine aminotransferase (OAT) (EC 2.6.1.13) deficiency that causes gyrate atrophy of the choroid and retina (GACR, MIM# 258870) is one of the diseases, which are caused by a deficiency of a protein predominantly expressed within the hepatocytes and therefore to achieve clinically relevant benefit hepatocyte protein expression needs to be restored. OAT is a pyridoxal phosphate‐dependent mitochondrial matrix enzyme. It catalyzes the conversion of ornithine into proline precursor pyrroline‐5‐carboxylate (P5C), and OAT deficiency is characterized by persistent hyperornithinaemia (10‐ to 20‐fold normal levels; Ginguay *et al*, 2017). A high concentration of ornithine has been shown to be toxic for retinal pigment epithelium. While there is a remarkable variability in the age of onset and disease progression, GACR typically presents in childhood with myopia and night blindness with a progressive loss in visual acuity, and leads to blindness in the fourth to sixth decades of life. Reducing plasma ornithine levels through a low‐protein or arginine‐restricted diet and vitamin B6 supplementation are the current treatment strategies; however, they can only decelerate the progression of the retinal symptoms with a variable individual response (Kaiser‐Kupfer *et al*, 2002; Montioli *et al*, 2021). Furthermore, the adherence to a complex diet is typically very poor, and therefore, there is still high unmet need for curative treatment.

In a recent study, Boffa *et al* (2022) administered intravenous retro‐orbital plexus injections of serotype 8 AAV (AAV8) vector expressing OAT under the control of a hepatocyte‐specific promoter (thyroxine‐binding globulin [TBG]) to two different OAT‐deficient mouse models (*Oat* ^*rhg*^ and *Oat* ^*Δ*^) at the age of 6 or 10 weeks.

The *Oat* ^*rhg*^ mice that have a later onset of retinal disease starting from 7 months of age were first injected with 1 × 10^13^ gc/kg of AAV‐OAT at the age of 6 weeks, which achieved a significant decrease in plasma and eye‐cup ornithine levels. Although ornithine concentration did not reach normal levels, it was still sufficient to improve the retinal pathology. In another experiment, *Oat* ^*rhg*^ mice were injected either with 1 × 10^13^ gc/kg or 3 × 10^13^ gc/kg of AAV‐OAT at a later age of 10 weeks when liver growth was completed, and thus, there was less chance of the loss of transgene expression with time. At 12 months postinjection, there was a dose‐related decrease in plasma ornithine concentrations besides lower ornithine concentrations in the eye cups, increased OAT activity in the liver, and almost normal appearance of retinal pigment epithelium (RPE) with an improved ERG response at both doses (Boffa *et al*, 2022).

The Oat null mice (*Oat* ^*Δ*^) have an earlier onset of retinal disease, develop retinal degeneration within the first 4 months of life, and were injected with 3 × 10^13^ gc/kg of AAV‐OAT at the age of 6 weeks. There was also a constant decrease in plasma ornithine concentrations and an improvement in retinal function and structure (Boffa *et al*, 2022).

Overall, these results highlight how the restoration of the liver enzyme expression led to the prevention of retinal disease in models of GACR (Fig 1). The researchers showed that the restoration of at least 10% of OAT activity in the liver was sufficient to achieve significant improvement in ornithine concentration and prevent retinal degeneration.

**Figure 1:**
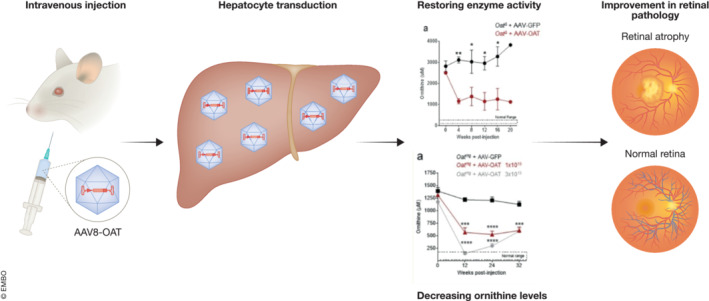
Preclinical study of AAV‐based liver‐directed gene therapy for gyrate atrophy of the choroid and retina due to ornithine aminotransferase deficiency

There are still challenges that need to be addressed when planning clinical translation. The consideration for the timing of vector administration should be carefully balanced between the desire to treat patients in late childhood or adulthood to achieve persistently high levels of OAT expression and earlier treatment to pre‐empt the onset of retinal degeneration.

OAT can compete with the urea cycle for ornithine pool, and increased OAT activity may cause the depletion of ornithine used in ureagenesis, which could lead to anormal protein degradation and toxic hyperammonaemia. Although there were no signs of defective ureagenesis in any of the injected mouse models, ureagenesis studies may need to be implemented as a safety endpoint in future clinical studies (Boffa *et al*, 2022).

In conclusion, the study represents a new exciting approach for treatment of an ocular disease with high unmet needs by using AAV‐based gene therapy targeting the liver to achieve metabolic control.
